# Regulation of Skeletal Muscle Function by Amino Acids

**DOI:** 10.3390/nu12010261

**Published:** 2020-01-19

**Authors:** Yasutomi Kamei, Yukino Hatazawa, Ran Uchitomi, Ryoji Yoshimura, Shinji Miura

**Affiliations:** 1 Graduate School of Life and Environmental Sciences, Kyoto Prefectural University, Kyoto 606-8522, Japan; 2 Ajinomoto Co., Inc., Kanagawa 210-8681, Japan; 3 Department of Health and Nutrition, Faculty of Health Management, Nagasaki International University, Nagasaki 859-3298, Japan; 4 Graduate School of Nutritional and Environmental Sciences, University of Shizuoka, Shizuoka 422-8526, Japan

**Keywords:** amino acid, skeletal muscle, PGC1*α*, exercise, energy expenditure, branched-chain amino acid (BCAA), leucine, *β*-hydroxy-*β*-methylbutyrate (HMB), *β*-aminoisobutyric acid (BAIBA), metabolic diseases

## Abstract

Amino acids are components of proteins that also exist free-form in the body; their functions can be divided into (1) nutritional, (2) sensory, and (3) biological regulatory roles. The skeletal muscle, which is the largest organ in the human body, representing ~40% of the total body weight, plays important roles in exercise, energy expenditure, and glucose/amino acid usage—processes that are modulated by various amino acids and their metabolites. In this review, we address the metabolism and function of amino acids in the skeletal muscle. The expression of PGC1*α*, a transcriptional coactivator, is increased in the skeletal muscle during exercise. PGC1*α* activates branched-chain amino acid (BCAA) metabolism and is used for energy in the tricarboxylic acid (TCA) cycle. Leucine, a BCAA, and its metabolite, *β*-hydroxy-*β*-methylbutyrate (HMB), both activate mammalian target of rapamycin complex 1 (mTORC1) and increase protein synthesis, but the mechanisms of activation appear to be different. The metabolite of valine (another BCAA), *β*-aminoisobutyric acid (BAIBA), is increased by exercise, is secreted by the skeletal muscle, and acts on other tissues, such as white adipose tissue, to increase energy expenditure. In addition, several amino acid-related molecules reportedly activate skeletal muscle function. Oral 5-aminolevulinic acid (ALA) supplementation can protect against mild hyperglycemia and help prevent type 2 diabetes. *β*-alanine levels are decreased in the skeletal muscles of aged mice. *β*-alanine supplementation increased the physical performance and improved the executive function induced by endurance exercise in middle-aged individuals. Further studies focusing on the effects of amino acids and their metabolites on skeletal muscle function will provide data essential for the production of food supplements for older adults, athletes, and individuals with metabolic diseases.

## 1. Introduction: Amino Acids and Health

Amino acids are compounds that contain an amino group (-NH2) and a carboxyl group (-COOH) and are components of proteins and materials for various bioactive molecules. As amino acids are known to be biologically safe, they are used for food and non-food purposes. Amino acids, and other food components, can be divided into those with nutritional, sensory, and biological regulatory functions. First, their nutritional function means that eating a sufficient amount of good-quality protein leads to an adequate source of essential amino acids. In contrast, eating proteins with an amino acid imbalance leads to a shortage of essential amino acids. Some plant proteins lack certain amino acids (i.e., lysine in cereals) that are necessary for animal growth; therefore, these amino acids are often added to certain foods to enrich their nutritional value [1,2]. Moreover, in individuals who are unable to eat protein, amino acid preparations are used for the nutritional management of pathological conditions. Second, with regard to the sensory function, some amino acids exhibit one or more of the five tastes (sweetness, sourness, saltiness, bitterness, and umami) [3] and are used to flavor processed foods. Third, amino acids have biological regulatory functions, for example, leucine increases the anabolism of muscle proteins [4,5], arginine has a vasodilation action and enhances immunity [6], and gamma aminobutyric acid (GABA) regulates blood pressure [7,8]. In addition to these three functions, amino acids can also be used as medical diagnostic tools and to predict the risk of various diseases, such as cancer, by measuring their levels in blood [9]. Thus, the effects of amino acids on health are a highly important issue. In this review, we discuss the roles of amino acids in the skeletal muscle.

## 2. Skeletal Muscle and Prevention of Metabolic Diseases

The skeletal muscle is the largest organ in the human body, representing ~40% of the total body weight, which stores energy in the form of proteins (amino acids). The skeletal muscle exhibits plasticity in response to the environment; proper exercise combined with adequate nutrition leads to muscle hypertrophy. Conversely, motor incapacity and aging cause muscle atrophy, which leads to decreased energy expenditure (obesity), decreased blood glucose uptake by the skeletal muscle and increased blood glucose levels (diabetes), and a lower quality of life [10]. In aged societies, such as those in developed countries, muscle atrophy suppression is important for health and longevity. Moreover, exercise has been shown to affect not only the skeletal muscle but also various other organs [11,12]. An understanding of the metabolism of the skeletal muscle during exercise and of the mechanisms of muscle atrophy is important for the prevention and treatment of metabolic diseases and muscle dysfunction.

## 3. PGC1*α* and Amino Acid Metabolism in the Skeletal Muscle

Most amino acids are metabolized in the liver. The branched-chain amino acids (BCAAs; valine, leucine, and isoleucine) are thought to be metabolized and utilized as energy sources in the skeletal muscle [13], as the expression of BCAA aminotransferase (BCAT2), an enzyme that catabolizes the first step of BCAA degradation, is high in the skeletal muscle and low in the liver. The branched-chain *α*-keto acid dehydrogenase (BCKDH) catabolizes the second reaction step of the BCAA degradation pathway [14,15].

Peroxisome proliferator-activated receptor-gamma coactivator 1α (PGC1α) is a coactivator of transcription factors, including nuclear receptors, and activates the expression of fatty acid oxidation genes [16]. PGC1α is also important for the regulation of mitochondrial biogenesis and the formation of muscle fibers (i.e., slow-twitch fiber formation) [17,18]. Moreover, PGC1α expression is increased by prolonged exercise; transgenic mice overexpressing PGC1α exhibited a high mitochondria content, slow-twitch muscle fiber formation, and increased continuous running time [19].

**Figure 1:**
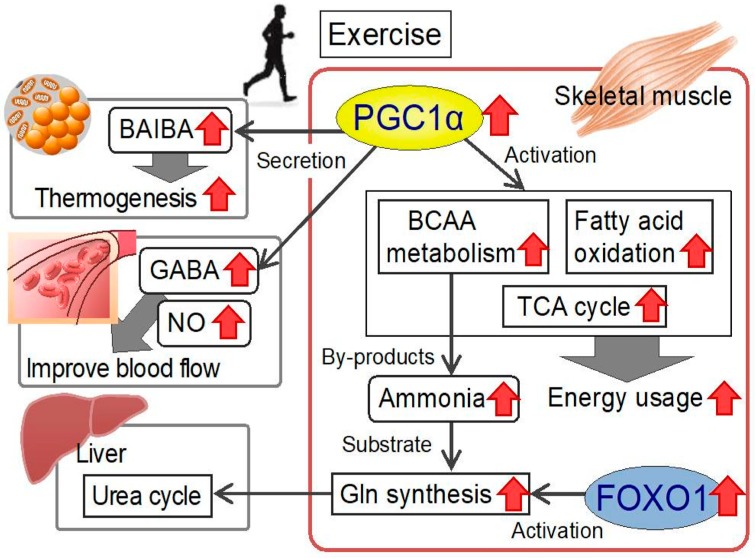
Metabolic changes in the skeletal muscle during exercise and amino acid-mediated interorgan effects. PGC1α expression in the skeletal muscle is increased by exercise. Increased PGC1*α* activates BCAA metabolism, fatty acid oxidation, and the TCA cycle and increases energy usage [20,24]. BCAA degradation leads to the formation of ammonia by-products. FOXO1 increases glutamine synthetase (adds ammonia to glutamic acid), resulting in the elimination of ammonia from the liver (urea cycle) [23]. In turn, exercise-induced PGC1*α* increases BAIBA, GABA, and arginine levels in the skeletal muscle [24]. BAIBA secreted from the skeletal muscle causes browning of white adipose tissue and increases thermogenesis [27]. GABA and arginine-derived NO may act on blood vessels and improve blood flow. Thus, in terms of preventing metabolic diseases, myokines are likely to be important, as myokines mediate the signaling of the favorable effects of exercise from the skeletal muscle to other organs. Ingestion of these amino acids as supplemental foods may improve human health. PGC1*α*, peroxisome proliferator-activated receptor *γ* coactivator 1-*α*; BCAA, branched-chain amino acid; TCA cycle, tricarboxylic acid cycle; FOXO1, forkhead box protein O1; Gln, glutamine; BAIBA, *β*-aminoisobutyric acid; GABA, *γ*-aminobutyric acid; NO, nitric oxide.

Previously, we conducted microarray analysis to ascertain the phenotypes induced by PGC1α. Bioinformatics analysis identified fatty acid metabolism, which had been described previously, and the BCAA metabolism pathway—a previously unrecognized pathway. In particular, the expression of BCAA metabolic enzymes, such as BCAT2 and BCKDH, increased. These data suggest that exercise-induced PGC1*α* expression activates BCAA metabolism (Figure 1) [20,21]. PGC1*α* also increases alanine synthesis via alanine aminotransferase in muscle cells [22].

During BCAA degradation, ammonia is produced from amino groups. Although ammonia is harmful to cells, there is no ammonia-clearance pathway in the skeletal muscle. We have previously shown that, during intense exercise, the forkhead box protein O1 (FOXO1) transcription factor upregulates the glutamine synthetase gene [23]. The resulting glutamine (via the addition of an amino group to glutamic acid) is likely to be transferred to the liver and used in the urea cycle [23]. We also conducted a comprehensive metabolomic analysis of metabolite changes in the skeletal muscle in PGC1α-transgenic mice. We found that the metabolites of the TCA cycle were increased in those animals [24]. Moreover, the metabolites of the purine nucleotide cycle and aspartate–malate shuttle, which are activated during exercise, were also increased. Thus, PGC1*α* is likely to use various substrates, including amino acids, to activate the TCA cycle; therefore, amino acids are used as a source of energy during exercise (Figure 1) [24].

Furthermore, PGC1*α* is reported to integrate the mammalian clock and energy metabolism [25]. Specifically, PGC1*α* stimulates the expression of transcription factors, Bmal1 and RevErb*α*, which regulate the circadian rhythm [25]. Recently, Dyar et al. reported that metabolomic analysis of skeletal muscle-specific Bmal1-knockout mice exhibited mostly altered blood amino acid levels during the circadian rhythm [26]. Skeletal muscle PGC1*α* and circadian transcription factors are likely to be important for muscle and systemic amino acid metabolism.

## 4. Amino Acids in the Regulation of Muscle Mass

**Figure 2:**
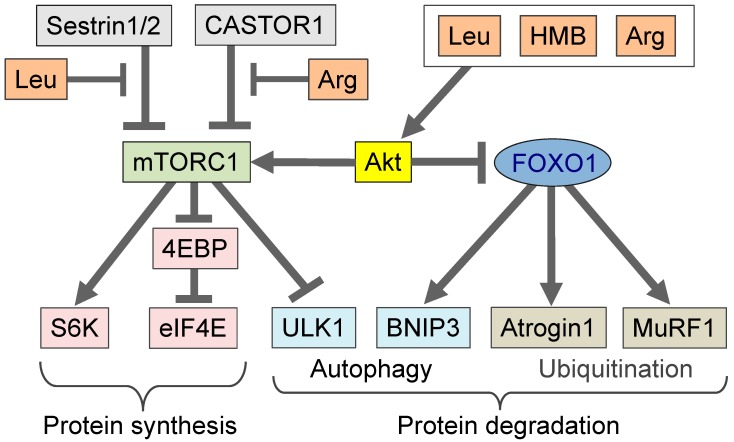
mTORC1 is activated by amino acids, such as leucine, HMB, and arginine. mTORC1 phosphorylates substrates, such as 4EBP and S6K, and increases protein synthesis. Moreover, in the presence of these amino acids, mTORC1 suppresses starvation signals, such as autophagy. Amino acids (leucine, HMB, and arginine) can activate Akt, leading to mTORC1 activation and FOXO1 suppression [28,34,35,36]. FOXO1 is a transcription factor that induces muscle atrophy. Suppression of FOXO1 transcriptional activity leads to decreased autophagy. Leucine interacts with Sestrin 1 or Sestrin 2 [5,32], and arginine interacts with CASTOR1 and activates mTORC1 [33]. The nature of the molecules involved in the amino acid-mediated pathway (e.g., the differences among leucine, HMB, and arginine) warrants further clarification. Leu, leucine; HMB, *β*-hydroxy-*β*-methylbutyrate; Arg, arginine; mTORC1, mammalian target of rapamycin complex 1; CASTOR1, cytosolic arginine sensor for mTORC1 subunit 1; FOXO1, forkhead box protein O1; S6K, S6 kinase; eIF4E, eukaryotic initiation factor 4E; 4EBP, eIF4E-binding protein; ULK1, unc-51 like autophagy activating kinase; BNIP3, Bcl-2 19 kDa interacting protein 3; MuRF1, muscle RING-finger protein-1.

Food proteins contain a high amount of BCAA (50% of essential amino acids and 20% of total amino acids from food) [13]. The BCAA leucine stimulates protein synthesis, and the molecular mechanism behind leucine as a nutritional-signaling molecule has been described [4,5]. The leucine-induced stimulation of protein synthesis activates the translation process (from mRNA to protein), and a molecular complex containing the eukaryotic initiation factor 4E (eIF4E) is important for the initiation of translation. During amino acid depletion and starvation, the eIF4E-binding protein (4EBP) binds to eIF4E to decrease the activity of the translation initiation complex and suppress translation initiation. Leucine activates the mammalian target of rapamycin complex 1 (mTORC1) kinase [28], which phosphorylates 4EBP, leading to its dissociation from eIF4E, the initiation of translation, and an increase in protein synthesis (Figure 2). In fact, we found that oral administration of leucine to mice increased 4EBP phosphorylation in the skeletal muscle [29], and the effect was mediated by BCKDH activity. Genetic deletion of the branched-chain α-keto-dehydrogenase kinase (BDK) [30], an inhibitor of BCKDH, or addition of fibrate, an inhibitor of BDK, increased BCKDH activity [31] and decreased cellular leucine levels and mTORC1 activity. These findings suggest that the regulation of muscle BCAA metabolism affects protein synthesis.

Leucine has been found to activate mTORC1 via Sestrin 2, i.e., binding of leucine to Sestrin 2 disrupts its interaction with GTPase-activating protein toward rags 2 (GATOR2), thereby activating the mTORC1 signaling pathway [5]. Meanwhile, Xu et al. reported that Sestrin 1, but not Sestrin 2, is highly expressed in the skeletal muscle and that leucine activates mTORC1 via Sestrin 1 [32]. In addition to leucine, arginine has also been reported to activate mTORC1 (Figure 2) [5]; arginine binds to cytosolic arginine sensor for mTORC1 subunit 1 (CASTOR1) and disrupts its interaction with GATOR2, thereby activating mTORC1 [33].

Because leucine and BCAAs stimulate protein synthesis, they are likely to contribute to the prevention of muscle atrophy. It has been reported that the administration of a BCAA-rich feed to middle-aged mice activated mTORC1 and suppressed atrophy [37]. In addition, the intake of a leucine-rich supplement by elderly people improved muscle mass, muscle strength, and walking speed [38], and older people who ingested BCAA exhibited enhanced muscle protein synthesis [39]. Furthermore, administration of BCAA was more effective than that of leucine alone for recovery from high-intensity resistance exercise [40]. Additionally, arginine administration resulted in increased protein synthesis [36]. Meanwhile, BCAA supplementation did not improve muscle recovery from intense resistance exercise in young adults [41]. Thus, BCAAs and arginine appear to be able to prevent and/or improve age-related muscle atrophy, i.e., sarcopenia.

Although the details of the mechanism are unclear, several studies have revealed that other amino acids can activate the mTORC1 pathway or increase muscle mass. For example, glycine activates mTORC1 in C2C12 myoblasts [42]. In a rodent atrophy model induced with total gastrectomy, the administration of BCAA combined with glutamine prevented muscle mass reduction [43]. In addition, administration of leucine alone or in combination with glutamic acid is beneficial for muscle growth during the fattening of pigs [44]. Dietary supplementation, with both arginine and glutamic acid, decreases the mRNA levels of genes involved in protein degradation in the skeletal muscle of pigs [45]. In addition to BCAAs, lysine also reportedly suppresses protein degradation in the skeletal muscle, e.g., the ingestion of lysine suppressed protein degradation in a mouse model of sarcopenia in aging individuals [46]. Therefore, ingestion of amino acids can modulate the degradation and synthesis of muscle proteins and may be used to suppress muscle atrophy related to undernutrition, disuse, and aging. BCAA and several other amino acids appear to be effective for the activation of mTORC1 and the increase of muscle mass.

## 5. *β*-hydroxy-*β*-methylbutyrate (HMB) in Comparison to Leucine

*β*-hydroxy-*β*-methylbutyrate (HMB) is a metabolite of leucine. Generally, leucine is metabolized to α-ketoisocaproic acid (KIC) by branched-chain amino acid aminotransferase 2 (BCAT2) in the skeletal muscle. Most of the KIC is converted to isovaleryl-CoA, and only 5–10% of KIC is converted to HMB in the liver [47]. HMB has been used as an ergogenic supplement to increase muscle mass and strength in humans. Several studies have revealed that HMB and leucine stimulate protein synthesis and reduce muscle protein breakdown. HMB and leucine increases the phosphorylation of FOXO1 and decreases nuclear FOXO1 levels, resulting in the downregulation of muscle atrophy-related muscle RING-finger protein-1 (MURF1) (Figure 2) [34,35]. Suppression of the FOXO1 pathway by HMB or leucine may prevent muscle atrophy, as FOXO1 is an important transcription factor in this process [48]. Similarly to leucine, HMB increases protein synthesis through the activation mTORC1. Treating myoblasts with HMB increases Akt phosphorylation [34] and, subsequently, activates mTORC1 signaling (Figure 2). To our knowledge, no report has described the activation of mTORC1 by HMB by Sestrin 2 or Sestrin 1. Thus, the signals produced by HMB to activate mTORC1 may be distinct from those of leucine (Figure 2).

To induce its anabolic effects, the HMB blood concentration must be above a certain threshold. In vitro studies have shown the effective HMB concentration to be 50 μM in cultured myocytes [34]. HMB ingestion (2.42 g) by participants was found to increase the plasma HMB concentration to 400 μM; however, ingestion of leucine (3.42 g) increased plasma HMB levels to 10 μM [49]. Therefore, endogenous HMB derived from the ingestion of leucine does not appear to be a major contributing factor in skeletal muscle anabolism. The detailed mechanisms underlying the actions of leucine and HMB (i.e., differences and similarities) in the skeletal muscle warrant further clarification.

## 6. Valine Metabolites

BAIBA stems from mitochondrial valine catabolism and is produced by the skeletal muscle during exercise [27]. Moreover, it was shown to communicate the beneficial effects of exercise from the skeletal muscle to other tissues and organs in an endocrine manner. BAIBA increases energy expenditure by activating the *β*-oxidation pathway of hepatic fatty acids, triggers the browning of white adipose tissue, is inversely correlated with cardiometabolic risk factors [27], and improves insulin resistance and inflammation in the skeletal muscle [50]. We previously reported that BAIBA reduces tumor necrosis factor (TNF)-*α*-induced expression of vascular cell adhesion molecule (VCAM)-1 in human umbilical endothelial cells, suggesting that BAIBA acts to prevent atherosclerosis by physical training [51].

There are two enantiomers of BAIBA in biological systems: L-BAIBA and D-BAIBA. L-BAIBA is generated from catabolic reactions of L-valine, whereas D-BAIBA is produced from thymine in the cytosol. Kitase et al. revealed that the production of L-BAIBA increases during muscle contraction, presumably because of intensive oxidation of L-valine [52]. L-BAIBA, secreted by skeletal muscle, has been shown to act on osteocytes; it diminishes the production of reactive oxygen species in mitochondria and protects osteocytes from apoptosis, preventing bone loss [52]. However, it is unknown whether systemic D-BAIBA levels are also affected by exercise or whether this regulation is specific to L-BAIBA.

Valine catabolic intermediate 3-hydroxy-isobutylate (3-HIB) stimulates fatty acid intake in the skeletal muscle and leads to insulin resistance, which may be due to the accumulation of intramuscular lipids [50,53]. Recently, Yoneshiro et al. reported that insufficient BCAA catabolism in brown adipose tissue led to obesity and diabetic phenotypes in mice [54]. BCAA catabolism in the skeletal muscle may also be related to obesity and diabetes. Sufficient metabolism of valine and BCAA may lead to decreased 3-HIB levels, which appears to be important for the prevention of obesity and diabetes.

## 7. Other Amino Acid Metabolites

Metabolomic analysis of PGC1*α*-transgenic mice revealed a marked increase in GABA, BAIBA, and amino acid metabolites [24]. GABA intake improves high blood pressure [7,8], as does regular exercise. Exercise-induced PGC1*α* expression increases GABA production and may contribute to the improvement of high blood pressure [24]. BAIBA and GABA are potential myokines that are secreted from exercised skeletal muscle and affect various other organs, and they may explain the exercise-mediated improvement of metabolic diseases via the interactions between muscles and other organs.

NO production improves blood flow. Increased endothelial nitric oxide synthase (eNOS) expression and muscle blood vessel formation were observed in PGC1*α*-transgenic mice [19]. This appears to be a situation in which the transfer of nutrients and oxygen during exercise is enhanced. Moreover, arginine supplementation in mice increases PGC1*α* expression [55], and arginine may activate PGC1*α*-mediated NO production. NO is produced from arginine; however, arginine taken orally is susceptible to degradation in the intestine, whereas the intake of citrulline, a precursor of arginine, increases blood arginine levels more effectively [56]. Thus, citrulline intake may improve blood flow through the skeletal muscle.

The amino acid, 5-aminolevulinic acid (ALA), is produced by ALA synthase (ALAS) and is important for the heme-biosynthesis process. PGC1*α* activates the expression of the ALAS gene in the liver [57]. We observed decreased ALAS expression in skeletal muscle-specific PGC1*α*-deficient mice, as assessed by microarray analysis [21]. ALA can control glucose metabolism in the skeletal muscle. Decreased ALA levels in mice and muscle cells attenuate mitochondrial function and cause impaired glucose tolerance and insulin resistance, which are recovered by ALA treatment [58]. In fact, cohort studies suggest that oral ALA administration can protect against mild hyperglycemia and may help prevent type 2 diabetes [58]. Thus, ALA may be a useful supplement to improve skeletal muscle function.

## 8. Metabolomic Analysis of Aged Skeletal Muscle

**Figure 3:**
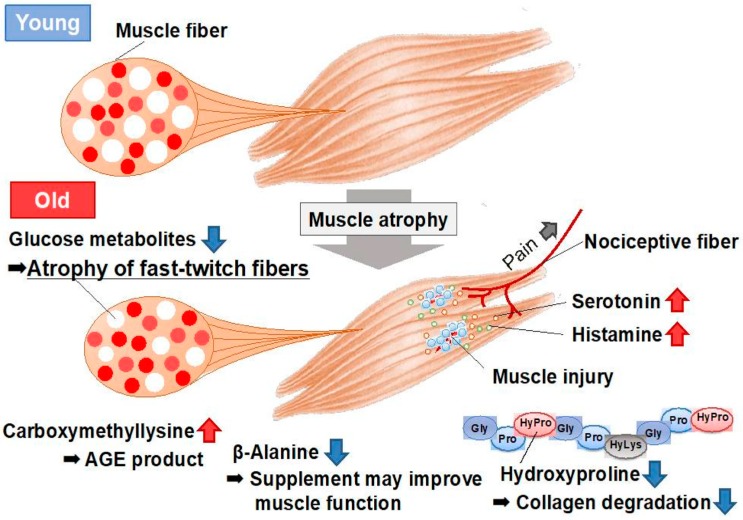
Metabolomic analysis of the skeletal muscles of young and aged mice [59]. Aged muscle exhibited atrophy, especially in fast-twitch fibers (white fibers), which was accompanied by decreased glycolytic metabolism. Increases in the levels of neurotransmitters (serotonin and histamine) were observed, which may indicate (or explain) muscle injury and pain in aged muscle. Carboxymethyllysine, which is an AGE product, also increased in aged muscle, whereas *β*-alanine markedly decreased. Supplementation with *β*-alanine may improve the muscle function in sarcopenia. AGE, advanced glycation end product.

Mouse models have facilitated the extensive study of sarcopenia, as mice have a lifespan of 2–3 years. To better understand the changes in the skeletal muscle that occur during sarcopenia, we conducted a metabolomics analysis of the skeletal muscle in young (8-week-old) and aged (28-month-old) mice using capillary electrophoresis with electrospray ionization time-of-flight mass spectrometry (CE-TOFMS), which can detect the various cellular water-soluble compounds related to amino acid metabolism (Figure 3) [59]. Glucose metabolic products, specifically fructose 1, 6-diphosphate and dihydroxyacetone phosphate, decreased in aged mice, possibly because of the decrease in the number of glycolytic muscle fibers. In turn, the levels of neurotransmitters, including histamine and serotonin, increased in the skeletal muscle of aged mice, as a probable consequence of the muscle injury associated with aging (Figure 3).

Changes in several amino acid-related metabolites were noted. The levels of hydroxyproline, a major component of collagen, decreased in aged mice (0.3-fold). Furthermore, mRNA levels of the collagen gene markedly decreased in the skeletal muscle of aged mice; therefore, the decrease in hydroxyproline levels may reflect the downregulation of the collagen gene in these models. Carboxymethyllysine, which is an advanced glycation end product (AGE) that accumulates during the aging process, was markedly increased in aged mice (5.0-fold), which is a likely phenotype of aging in these animals. In addition, the levels of carboxymethyllysine correlate with vascular diseases and arteriosclerosis in diabetic patients. S-Adenosylmethionine donates methyl groups to DNA and proteins, and the levels of global genomic DNA methylation increase in the skeletal muscle in response to aging. The increased S-Adenosylmethionine levels observed in aged muscle may be involved in the process of DNA methylation [59]. In addition, the level of *β*-alanine decreased in the skeletal muscle of aged mice (0.6-fold) (Figure 3). The literature reveals that *β*-alanine supplementation increased the physical performance and improved executive function induced by endurance exercise in middle-aged individuals [60]. *β*-Alanine supplementation may be useful for improving muscle function in cases of aged sarcopenia with reduced *β*-alanine. Further analyses focusing on the metabolites observed to be altered in that study, including amino acids, will provide data essential for understanding aging muscles.

## 9. Closing Remarks

Amino acids are critical for human health. In this review, we discussed the roles of amino acids in the skeletal muscle and the organs that interact with it. We described the relationships between the exercise-activated transcription regulator PGC1*α* and amino acids. In addition, we discussed the changes in amino acid metabolites during skeletal muscle aging. Clearly, BCAA and various other amino acids and their metabolites play important roles in the skeletal muscle. Further study of amino acids, especially in the skeletal muscle, will continue to benefit preventive medicine and health sciences.
